# Dr. Andrew Kamerling

**Published:** 1918-02

**Authors:** 


**Dr. Andrew Kamerling,** Buffalo, died Dec. 6, age 78. He graduated from the University of Buffalo in 1866.

